# *Case of* Gun-Shot Wound

**Published:** 1803-02-01

**Authors:** John King

**Affiliations:** Surgeon, of Bath


### *Case of* Gun-Shot Wound; *communicated by* Mr. John King, *Surgeon, of Bath.*

ON the evening of the 5th of November, 1802, Jacob Robbins, son of Mr. Robbins, baker, at Walcot, had the misfortune, whilst a spectator at a bonfire, &c. to stand parallel to a loaded gun-barrel, which was fixed in the ground, and when fired off, its contents struck on the young man's face, and produced a wound of a lacerated description. In this state, he was immediately brought to me, very languid from hæmorrhagy of the maxillary and other arteries, yet not alarming on that account, so as to demand any prompt application by ligature, or powerful styptic.

On inspecting the wound, I found it very deep, reaching high up towards the orbitar portion of the os frontis, and scaling along the nasal internal surface of the maxillary and palate bones, breaking down the vomer and crashing a very large portion of the os ethmoides.

The integuments were lacerated in the form of a triangle, commencing at the transverse frontal suture, and extending in a line direct to the margin of the upper lip; then proceeding obliquely from the frontal suture across the maxilla towards the angle of the lower jaw.

Another laceration was directed from the adipose pillar which sustains the septum nasi in a parallel line to the middle of the cheek; and part of the substance of the integuments were shot away, together with the cartilage which constitutes the septum.

The nasal process of the os maxillare was fractured, the orbitar process was cracked and loosened, and yet the eye was not in the least injured; not even did it assume in-

 flammation flammation, though an epiphora, the consequence of inflammation in the lacrymal canal, ensued. The spines of the maxillary bones, which with the vomer, form part of the nasal division, was broken down, and the square part of the maxillary basis on one side was depressed, but not very importantly.

The sinus, or Highmore's antrum as it is termed, was laid completely open, and portions of bones which form that cavity were taken away; part became carious, which, with the sloughing of its inner membrane, afforded a most offensive discharge, distilling through the nostrils, and behind the arch of the palate, into the throat and mouth, so that the surrounding bones, which assist in the formation of the sinus, were also shaken; notwithstanding, no inflammation invested the alveolar processes of the maxilla, only just enough to excite the pain of distension; indeed, it was a matter of surprize that neither the teeth nor alveolar processes were displaced, though the roots of the teeth were exquisitely sensible from the proximity of the injury inflicted, and the rupture of nervous parts. In the destruction offered to these parts, it was impossible for the os spongiosum inferius to escape, and likewise the os spongiosum superius, in its inferior part; therefore an obstruction in the lacrymal canal was the first thing to be expected, as the lower extremity of that canal must have been destroyed.

Thus the lateral cartilages of the nose, the os nasi, the nasal portion of the maxillary bone, and the cartilage constituting the septum nasi, were extruded, partly inflected back on the cheek, and partly sinking down over the mouth and chin, exposing to view the whole cavity from the internal nasal surface of the ossa maxillaria and palatina to the cribriform cells of the ethmoid bone, so that the os ethmoides was very materially injured. The ossa palati, and the palatine portions of the maxillary bones appeared to have suffered chiefly from contusion, as an ecchymosis was apparent on the concave surface of the palate.

In examining the excavation produced by the breaking down of so much solid parts, my attention was directed to the removal of every loose portion of bone and cartilage; after these were separated and extracted, I approximated, by means of the interrupted suture, (supported by strips of adhesive plaster) all the integuments that were capable of adjustment, so as to give something of form to this "rudis indigestaque moles;" after which I passed a bougie into each nostril, to maintain the ordinary passages, and to form a sort a sort of conduit for blood and matter. The bougies being doubled at the external extremities, were caused to rest on the maxilla superior over the incisores teeth, which served to keep the nose elevated, and to dilate the nostrils.

I had nothing to fear, however, from the future confinement of matter, as that would in part easily escape behind the palate into the mouth and throat, and in part by the nostrils.

I had in former instances of lacerated wounds, (where the contusion of surrounding parts was great, and the pain considerable) experienced so much the benefit of cooling applications in the first instance, that I resolved to apply compresses of lint soaked in the aq. litharg. acet. c. for the first twelve hours; the re-action in such cases being very great and oftentimes injuriously so, destroying all the good to be expected from sutures.

The re-action is gently obviated by these means, yet sufficient excitement is allowed to produce adhesive and suppurative inflammation; thus much time is saved in the future regeneration of solids, which a too enlarged suppuration always destroys.

The union by the first intention, is never more desirable than in wounds of the face, yet as so much destruction of parts was effected, it could hardly be expected throughout the course of so many sutures as were made, yet the parts united, beyond our hopes.

The use of cooling applications in lacerated wounds is never to be persevered in, whenever the re-action on the parts becomes tolerant, when the fear of impending gangrene is subsided, when the pain diminishes, and the tumefaction is not excessive, and the heat and redness are moderate. Whenever these circumstances occur, or promise to occur, the suppuration is then to be forwarded by gently stimulant applications, and by the accumulation of heat in the parts; with this intention a warm poultice covered with a little ungt. picis, is very proper; and where the solution and separation of membrane and cartilage are to be expected, and consequently much offensiveness of discharge, a few drops of spt. terebinth. may be mixed with it. By these applications, aided by the use of strong antiseptic fomentations, composed of camomile, wormwood, poppy heads, &c. frequently renewed, a kindly suppuration ensues; but these observations may perhaps be thought too much in detail.

On enquiry respecting the faculty of smelling, as so much of the Schneiderian membrane was torn, so the olfactory factory power was lost; but as nervous parts recruit and regenerate, as well as muscular, that sense will no doubt in some degree be repaired.

From the violence offered to the bones of the nose, from their continuity with those of the head, it was to have been expected that some derangement might have arisen to the function of the sensorium, especially as the ethmoid plate, with the caput gallinaginis, are in immediate proximity, and serve to sustain the anterior lobes of the brain, yet no symptoms of concussion took place for a moment.

From the sieve-like structure of the os ethmoides, we may reasonably induce, why no disturbance occurred to the senses; had this bone been of a compact solid texture, the shock must have been participated by the brain; but, fortunately, the resistance to be overcome, by the force applied, was not great, and that part of its force was likewise exhausted on the nasal process of the maxillary bone, as well as on the arch of the nose; and as the anterior nasal suture was split in two, so the lateral integuments of the nose were surprizingly spared, a very favourable circumstance for the adjustment of the features.

It is evident from the destruction of so much solids, that the void occasioned was very considerable; and from this circumstance, as there was at present no abolition of the senses, I augured that there would be none in future, as any resistance by compression was removed from the nature of the accident. As the arterial ramifications, which are distributed to the bones of the nose, and enter their substance, were shortened at their principal trunks, therefore desquamations and a solution of some portions of bone which yet remained, must be the consequence; but having extracted all the loose fragments, I did not expect any considerable exfoliations, but on the tenth day I extracted a loose portion of the os ethmoides, where it ascends upwards to the cribriform plate; and from the acute pain always attendant on the use of the probe, the olfactory nerves were no doubt exceedingly irritated, or even ruptured.

Notwithstanding the destruction to the nasal process of the maxillary bone, to the orbitar portion which was elevated above the circumference of the orbit, to the inferior os spongiosum, and therefore to the ductus nasalis, so as to induce considerable inflammation in that canal, yet, on examination per nasum, and also by a fine probe insinuated into the inferior punctum, I was convinced that the the lacrymal canal, though shortened in its passage, from its inferior extremity being torn, yet that, on the subsidence of the inflammation, the tears would again resume their natural course, we should have expected from the continuity of the os unguis with the nasal process of the maxillary bone, as well as from its lateral connection with the os spongiosum superius, that the os unguis must have been sacrificed, but the protection which the superior and inferior part of the orbit afforded, and the strong adhesion which the *membrana sacculi lacrymalis* acquires, by its intimate attachment to that slender bone, served to support it in its situation amidst surrounding demolition.

As the vomer and nasal lamella of the ethmoid bone serve to sustain the maxillary and palate bones, and from the concussion which the maxillary basis received, there will no doubt remain, for some time, a considerable impediment to the mastication of hard and solid food; but as the parts consolidate more and more, fresh exertions, and the effect of habit, will in time remedy that evil.

At the end of three weeks from the accident I found the bone denuded and sonorous, just over the roots of the incisor teeth; and by assisting its desquamation with the point of the probe, it soon came away.

In regard to the epiphora, the occlusion of the eye-lids continued for a month, owing to the diffusion in the cells of the eye-lids, and the weeping was likewise *not constant*. The lacrymal sac was exceedingly distended, so as indeed to threaten suppuration, and the secretion in the sac becoming very thick, served as a mucus plug to the lacrymal ducts, whereby the puncta were stopped up; but by frequent pressure with the finger on the sac, and irritatives to the pituitary membrane, the contents at last made their way through the nasal duct, (and continued so to do during the cure) by the side of the maxillary bone.

From the fracture of the maxillary bone, in its nasal and orbitar portions, the fossa rose a little out of the circumference of the circle; but as each part still enjoys a relative correspondence, so the epiphora will soon decline, and, indeed, was quite recovered, till the patient took cold, on his returning to his accustomed occupation, which he was able to do in the sixth week from the accident.

From the detail of this case, it appears to me, that cooling applications in the first instance, though to an extensive lacerated wound, had the desired effect of obviating too strong a re-action on the parts, and therefore, in all probability, prevented a suppuration of the lacrymal sac, sac, and perhaps a genuine and permanent fistula lacrymalis; but as the animal vitality is weak in the integuments of the nose, therefore in so lacerated a wound, the re-action must for a short time, be just moderated to prevent its destructive powers. As soon as the first re-action is managed, there is not much to be apprehended from any succeeding ones, for the parts are afterwards disposed to meet the increased excitement. But as in all osseous and cartilagenous parts, the circulation is slow, so to produce a speedy cure, it is necessary to renew frequent local impulsions, by stimulating the system and the parts themselves; by so doing, the arterial ossific ramifications dislodge the parts, destined to separate, and therefore very speedily produce a consolidation of the læsed parts.

Perhaps the reader will think I have detained him too long, therefore I shall only add, that the medicine our patient used during the whole cure, after the first twenty-four hours, was the following, viz.

℞. Extr. cinchon. flav. ℈ij. ad ʒj vin. rhab. ℥ss. aq. menth. pip. ℥vijss. ft. mist. cujus sumt. æger totam omnibus 24 horis.

And that the only local stimuli, after the separation of unsound parts was effected by suppuration, were warm water forced from the nose into the throat and mouth at every dressing, and the ungt. picis with spt. terebinth. as the dressings themselves.

*Bath, January* 13, 1803.

